# Clinical significance of *HRAS* and *KRAS* genes expression in patients with non–small-cell lung cancer - preliminary findings

**DOI:** 10.1186/s12885-021-07858-w

**Published:** 2021-02-06

**Authors:** Milena Pązik, Katarzyna Michalska, Marta Żebrowska-Nawrocka, Izabela Zawadzka, Mariusz Łochowski, Ewa Balcerczak

**Affiliations:** 1 Laboratory of Molecular Diagnostics and Pharmacogenomics, Department of Pharmaceutical Biochemistry and Molecular Diagnostics, Cathedral of Laboratory and Molecular Diagnostics, Medical University of Lodz, Muszynskiego 1, 90-151 Lodz, Poland; 2 Department of Thoracic Surgery, Memorial Copernicus Hospital, Medical University of Lodz, Lodz, Poland

**Keywords:** Lung cancer, NSCLC, *HRAS*, *KRAS*, Expression

## Abstract

**Background:**

The *RAS* family protooncogenes, including *KRAS*, *NRAS* and *HRAS*, encode proteins responsible for the regulation of growth, differentiation and survival of many cell types. The *HRAS* and *KRAS* oncogene mutations are well defined, however, the clinical significance of *RAS* expressions in non–small-cell lung cancer (NSCLC) is still uncertain.

**Methods:**

A total of 39 whole blood samples of NSCLC (the investigated group), collected at three points of time: at the time of diagnosis, 100 days and 1 year after the surgery as well as 35 tissue samples obtained during the surgery were included in this study. *HRAS* and *KRAS* genes mRNA expression were assessed using quantitative real-time polymerase chain reaction techniques.

**Results:**

Increased relative *HRAS* mRNA level in blood was found significantly more frequently in the group of smokers (*p* = 0.008). Patients with squamous cell carcinoma subtypes of NSCLC were more likely to show an overexpression of *HRAS* gene in blood, but not statistically significant (*p* = 0.065). In tumor tissue overexpression of *HRAS* gene was associated with adenocarcinoma subtype (*p* = 0.049). No statistically significant associations were found for the expression of *KRAS* with any clinicopathological parameters, except the age of patients, within the study. There were no differences between the relative *HRAS* and *KRAS* genes expression levels in blood samples taken from the same patients during the 3 observation points, as well as between blood collected from patients before surgery and tissue samples obtained during operation.

**Conclusion:**

The potential associations between high *HRAS* expression levels, age, smoking status and histological type of cancer were observed, which emphasizes the need for further study of the *RAS* family. Therefore, subsequent research involving larger numbers of patients and a longer follow-up, as well as multicenter study are necessary to confirm our findings.

**Supplementary Information:**

The online version contains supplementary material available at 10.1186/s12885-021-07858-w.

## Background

Lung cancer is a significant worldwide health problem, accounting for more than 1.8 million new cases estimated in 2012 and 1.6 million cancer-related deaths annually [1]. The two main types are: small cell lung cancer (SCLC) and non-small-cell lung cancer (NSCLC). NSCLC comprises about 85% of all lung cancer cases, which have totally different etiology and treatment options [2]. NSCLC mainly includes squamous cell carcinoma, adenocarcinoma and large cell carcinoma. When patients are diagnosed in the early stages of NSCLC, the survival rates are relatively higher after a surgical resection. In patients with an untreated metastatic NSCLC present an overall survival (OS) rate in 1 year of only 10%, with a median survival of around 4 to 5 months [1].

Over the years, many genes/proteins have been discovered to play a key role in carcinogenesis [3]. RAS proteins are among the most commonly mutated cancer causing agents in humans and remain a difficult pharmaceutical target [4]. Members of the RAS family are low molecular weight monomeric, GTP-binding proteins that play a crucial role as a major component of cellular networks controlling various signaling pathways: growth regulation, proliferation, survival, differentiation, adhesion, cytoskeletal rearrangements, motility and cell survival [5, 6]. Previous research has improved the understanding of the structure, processing and signaling pathways of RAS in cancer cells and opened up new avenues for inhibiting RAS function [4, 5]. Abnormally activated RAS proteins regulate the function of major signaling pathways involved in the initiation and development in one-third of human cancers [3, 7, 8]. RAS proteins act as a cellular switch that is turned on by extracellular stimuli, resulting in the transient formation of an active, GTP-bound form of RAS that activates various signaling pathways which regulate basic cellular processes.

*RAS* genes family comprise of three *RAS* protooncogenes such as *HRAS*, *KRAS* and *NRAS* encoded four isoforms: *NRAS, HRAS, KRAS4A* and *KRAS4B*, the last two arising from alternative splicing variants of the *KRAS* gene [4]. The irreversible changes in the genetic content of a cell are a major cause of carcinogenesis as it can modulate both gene expression and the function of proteins involved in the regulation of cell growth and differentiation [3, 5]. *RAS* mutations have quite different patterns and vary with cancer. An oncogenic alteration in *KRAS* gene is the most frequent in pancreatic cancer, colorectal cancer and lung cancer, while mutated *HRAS* is the most common in dermatological, head and neck cancers. The *NRAS* mutations are often detected in hematological malignancies [3]. Since *RAS* genes are among the earliest mutated genes in various cancers, understanding how these mutation patterns arise can help not only understand how the cancer begins, but also the factors influencing the event that impact prevention and cancer treatment [7]. Till now, two general approaches have been explored for inhibiting RAS activity with small molecules: compounds that bind directly to RAS protein and inhibitors of the enzymes involved in the post-translational modifications of RAS [4].

The molecular development of NSCLC is initiated by the activation of oncogenes or the inactivation of tumor suppressor genes. Mutation of *KRAS* gene in lung cancer is more frequent than *NRAS* and *HRAS* and is often associated with poor prognosis and worse therapeutic outcome. Lung adenocarcinoma, the most common histological subtype of non-small-cell lung cancer (NSCLC), often carries a *KRAS* mutation with 20–50% frequency, followed by squamous cell carcinoma, subtype of NSCLC [3].

The present study was designed to determine the potential significance and the clinical relevance of two *RAS* gene family isoforms (*KRAS* and *HRAS*) and their mRNA expression levels in the group of non-small-cell lung cancer patients. Majority of previous scientific findings have focused on the mutation profiles of NSCLC patients or evaluation genes expression in lung cancer tissue. This research was aimed at analyzing the relative levels of *KRAS* and *HRAS* genes mRNA expression in whole blood samples in parallel with tumor tissue and, what is an innovative approach, at three points of time during the patients’ observation.

## Methods

### Materials

This retrospective study included 39 patients (32 male and 7 female) with NSCLC from the Department of Thoracic Surgery of the Medical University of Lodz, N. Copernicus Regional Specialist Hospital in Lodz, Poland. Patients with surgically treated NSCLC were included in the experiment. Tissue samples were obtained during the surgery (complete tumor resection – R0 in all of patients) and histopathologically confirmed as NSCLC, while peripheral blood samples were taken at three points of time during the disease and treatment process. The exclusion criteria were small cell lung cancer and carcinoid recognized after histologic examination. The investigation was in accordance with the Declaration of Helsinki, the Good Laboratory Practice rules and was approved by the Ethical Committee of the Medical University of Lodz (No: RNN/87/16/KE, RNN 85/20/KE). All patients provided a written informed consent before their inclusion in the study.

Thirty-nine blood samples at time of the diagnosis of cancer, 37 blood samples 100 days after the surgery, 26 blood samples 1 year after the surgery and 35 tissue samples collected during the surgery procedure were included in the analysis. These differences between the number of groups resulted from causes such as the death of patients between consecutive examination or losing patients from observation. In 13 cases adjuvant chemotherapy was included after the surgery (carboplatin + gemcitabine 3, cisplatin + vinorelbine 10). At the stage of diagnosis, the cohort was not evaluated for *KRAS* gene status. Clinical data, including sex, age at diagnosis, tumor histology, clinical stage, and smoking history, were collected from patients’ medical records. The following criteria were used to classify smoking status: non-smokers were defined as those who had declared never smoking and smokers, who had from 20 to 50 pack-years smoking history. The detailed clinicopathological and laboratory characteristics each of NSCLC patients are shown in Additional file 1.

### RNA isolation

RNA was successfully isolated from whole blood samples and frozen tissue samples using a Total RNA Prep Plus Minicolumn Kit *(A&A Biotechnology, Poland)* according to the manufacturer’s protocol. The purity and the concentration of RNA in samples were measured nanospectrophotometrically. The concentration in all samples derived from patients suffering from NSCLC was adjusted to 0.05 μg/μl. Until the analysis, the RNA samples were stored at − 80 °C.

### Reverse transcription

RNA samples were transcribed into cDNA using a High Capacity cDNA Reverse Transcription kit *(Applied Biosystems, USA)*, according to the manufacturer’s instructions. The thermocycling reverse transcription parameters were as follows: 25 °C for 10 min, then 37 °C for 120 min and 85 °C for 5 min. Until the analysis, the cDNA samples were stored at − 20 °C.

A housekeeping *GAPDH* gene, encoding glyceraldehyde-3-phosphate dehydrogenase, was used as reference gene. The samples with the presence of PCR product for the *GAPDH* gene (145 bp) were used in the following analysis.

### PCR amplification

The qualitative assessment of the obtained cDNA was performed by means of PCR amplification of the *KRAS* and *HRAS* genes JumpStart REDTaq ReadyMix *(Sigma-Aldrich, USA)*.

PCR reactions were performed in a volume of 20 μL, components of the PCRs for both genes were the same: 0.7 μl of each primer at a concentration of 10 μM for *KRAS* (forward GCCTGCTGAAAATGACTG, reverse TCCTGTAGGAATCCTCTATTG) and for *HRAS* (forward CACGGAAGGTCCTGAGGGG, reverse GCCTGGCCCCACCTGTG); 10 μl of Reaction Mix, 0.2 μl of 25 mM magnesium chloride solution and 1 μl of template.

PCR amplification was set at an initial 96 °C for 1 min and then, for both of genes, 34 cycles of 94 °C for 50 s, 57 °C (*KRAS*) and 58 °C (*HRAS*) for 50 s, 72 °C for 1 min, and the final extension at 72 °C for 7 min. The annealing temperature was standardized by gradient PCR at 56 °C–61 °C for both genes. After the reaction, PCR products 134 bp for *KRAS* and 276 bp for *HRAS* were evaluated during electrophoresis in 2% agarose gel to assess the quality of cDNA.

### Real-time PCR

Relative mRNA expressions level of *HRAS* and *KRAS* genes were analyzed using a quantitative real time-polymerase chain reaction (qRT-PCR) on The CFX Connect Real-Time PCR Detection System *(Bio-Rad Laboratories).* The reaction mixture contained: 5 μl iTaq Universal SYBR Green Supermix *(Bio-Rad Laboratories)*, 0.3 μl of a 10 μM solution of each primer specific for the investigated genes, 3.4 μl of distilled water and 1 μl of template up to 10 μl final volume. The qRT-PCR reactions for each sample were performed in triplicates. The same primer set was used for the qualitative analysis. A negative control without template was also added in a triplicate. The real-time PCR conditions were as follows: 10 min 95 °C primary denaturation, 40 cycles of 95 °C for 30 s, 58 °C for 1 min and 72 °C for 1 min. The mean of the obtained Ct values for three genes was counted.

The ΔΔCt method was used to calculate relative expression level changes in genes [9]. The mean Ct values for the *KRAS* and *HRAS* genes and *GAPDH* as a reference gene achieved for all tested samples were assumed as a calibrator. In subsequent reactions, specific amplification was verified using the melting curve analysis.

### Statistical analysis

The statistical analysis was performed using the Statistica 13.1. *(StatSoft, Inc., Tulsa, OK, USA*).The obtained quantitative data were verified with a normal distribution using the Shapiro-Wilk test. Due to the lack of conformity with normal distribution, a comparative statistical analysis was performed using the nonparametric U-Mann Whitney test. The Friedman test and Kendall’s W test were used to verify the differences between the relative level of *KRAS* and *HRAS* genes expression between blood samples collected at three points of time. The Wilcoxon test was applied to compare genes expression in blood samples obtained before the surgery with tissue samples taken during the surgery. In all conducted tests a *p* value of < 0.05 was assumed as statistically significant.

## Results

### Patient characteristics

The investigated group consisted of whole blood samples collected at the 3 points of time: 39 samples at time of diagnosis, 37 samples at 100 days after the surgery, 26 samples taken 1 year after diagnosis and 35 tissue samples obtained during the surgery procedure.

A total of 23 Polish patients with squamous cell carcinoma subtypes of NSCLC and 16 Polish patients with adenocarcinoma subtypes of NSCLC were enrolled. Investigated group included 32 (82%) men and 7 (18%) women. There were 14 (36%) non-smokers and 25 (64%) cigarette smokers, the median age was 68 years (ranging between 54 and 82 years). In this group 29 (74%) were at the G2 tumor histological grade, 8 (21%) at G3 tumor grade and 3 (8%) at G1. All patients underwent complete tumor resection and for 26 of them (66.7%) no other treatment was administered. 13 (33.3%) patients at stage IIB and IIIA received chemotherapy.

Sex, smoking status, histological type and pathologic stage distribution of the investigated group diagnosed as non-small-cell lung cancer with histopathological examination as well as white blood cells, red blood cells and platelet count are listed in detail in Table 1. In addition, the Neutrophil to Lymphocyte Ratio (NLR), the Lymphocyte to Monocyte Ratio (LMR) and the Platelet to Lymphocyte Ratio (PLR) were calculated.

**Table 1:** Clinicopathological and laboratory characteristics of patients with NSCLC

Parameter	N	%	Median	Range
Total	39			
Sex				
Male	32	82		
Female	7	18		
Smoking				
Smoker	25	64		
Non-smoker	14	36		
Histological type of cancer				
Adenocarcinoma	16	41		
Squamous cell carcinoma	23	59		
Grade of histological malignancy [G]				
G1	3	8		
G2	29	74		
G3	8	21		
TNM stage				
IA1	1	3		
IA2	7	18		
IB	11	28		
IIA	7	18		
IIB	5	13		
IIIA	8	21		
Chemotherapy				
No	26	66.6		
Yes	13	33.3		
WBC (× 10^9^/l)			9.65	6.02–19.39
RBC (× 10^12^/l)			4.61	3.54–5.84
PLT (×10^9^/l)			299	167–961
NLR			3.88	0.97–20.39
LMR			2.2	0.62–5.87
PLR			154	61–976

*Abbreviations*: *NLR* Neutrophil to Lymphocyte Ratio, *LMR* Lymphocyte to Monocyte Ratio, *PLR* Platelet to Lymphocyte Ratio, *TNM* Tumor-node-metastasis, *WBC* White blood cells, *RBC* Red blood cells, *PLT* Platelets

### Qualitative expression of the KRAS and HRAS genes in blood patients with NSCLC

All of the samples revealed the presence of *GAPDH* gene expression using quantitative PCR. 39 samples demonstrated the presence of *KRAS* and *HRA*S gene expression at the time of the diagnosis. 100 days after the surgery, 25 out of 37 samples revealed a qualitative *KRAS* gene expression and all of them presented a qualitative *HRAS* gene expression. One year after the surgery, 17 out of 26 samples were *KRAS* positive and 21 out of 26 samples were *HRAS* positive. Other samples showed no expression of the investigated genes at the second and third point of time during observation. The quantitative analysis indicated that genes expression of the tested *KRAS* and *HRAS* relative to the reference gene *GAPDH* were highly varied.

### Quantitative expression of the KRAS and HRAS genes in blood patients with NSCLC

All samples with the presence of *GAPDH* gene expression were included into the quantitative analysis. Relative *KRAS* and *HRAS* mRNA expression levels was determined using qRT-PCR in all 39 blood samples that were available for the analysis. At the time of the diagnosis, the median *KRAS* mRNA expression was 1.055 (ranging between 0.075–5.339), while the median *HRAS* mRNA expression was 1.153 (ranging between 0.122–4.376). The associations of median relative *KRAS* and *HRAS* mRNA expression (R-value) with clinicopathological features of NSCLC patients are summarized in Table 2.

**Table 2:** Relative expression levels of *KRAS* (A) and *HRAS* (B) mRNA in blood of NSCLC patients, compared to clinicopathological and demographical features

		Relative ***KRAS*** expression level in blood				
Relative ***KRAS*** expression level		N	Median	Minimum	Maximum	***p***-value
A						
All cases		39	1.0550	0.0753	5.3395	
Cigarettes	No	14	0.9968	0.0753	2.6566	0.849
	Yes	25	1.1025	0.4669	5.3395	
Sex	Women	7	1.0137	0.5820	2.6566	0.756
	Men	32	1.0787	0.0753	5.3395	
Age	<= 68	22	1.0787	0.2558	5.3395	0.403
	> 68	17	1.0137	0.0753	2.0498	
Histological type of cancer	Squamous	23	1.0137	0.4669	3.1129	0.558
	Glandular	16	1.0787	0.0753	5.3395	
TNM stage	IA1 and/or IA2 and/or IB	19	1.0137	0.0753	5.3395	0.603
	II A and/or II B and/or IIIA	20	1.0787	0.4669	2.6566	
Grade of histological malignancy [G]	G1 and/or G2	31	1.1025	0.0753	5.3395	0.339
	G3	8	0.9094	0.2558	2.6566	
Chemotherapy	No	26	0.9968	0.0753	5.3395	0.205
	Yes	13	1.1025	0.8031	2.6566	
B						
All cases		39	1.1532	0.1223	4.3762	
Cigarettes	No	14	0.8188	0.1223	1.8282	0.008
	Yes	25	1.3125	0.3291	4.3762	
Sex	Women	7	0.9777	0.1397	1.9223	0.596
	Men	32	1.1868	0.1223	4.3762	
Age	<= 68	22	1.2519	0.1397	4.3762	0.342
	> 68	17	0.9777	0.1223	1.9636	
Histological type of cancer	Squamous	23	1.2358	0.3291	4.3762	0.065
	Glandular	16	0.8735	0.1223	1.8282	
TNM stage	IA1 and/or IA2 and/or IB	19	1.1532	0.1223	1.9636	0.683
	II A and/or II B and/or IIIA	20	1.1260	0.1397	4.3762	
Grade of histological malignancy [G]	G1 and/or G2	31	1.1532	0.1223	2.5474	0.903
	G3	8	1.1747	0.1397	4.3762	
Chemotherapy	No	26	1.1868	0.1223	4.3762	0.333
	Yes	13	0.8922	0.1397	1.7389	

Initially, the examined population was divided into two groups according to age: 22 people under 68 years old and 17 people aged over 68 years of age. No statistically significant differences were observed between relative *KRAS* and *HRAS* gene expression levels and patients age (*p =* 0.403 and 0.343, respectively).

No statistical differences were also found for relative *KRAS* and *HRAS* gene expression levels between women and men (*p =* 0.756 and 0.596, respectively).

**Fig. 1:**
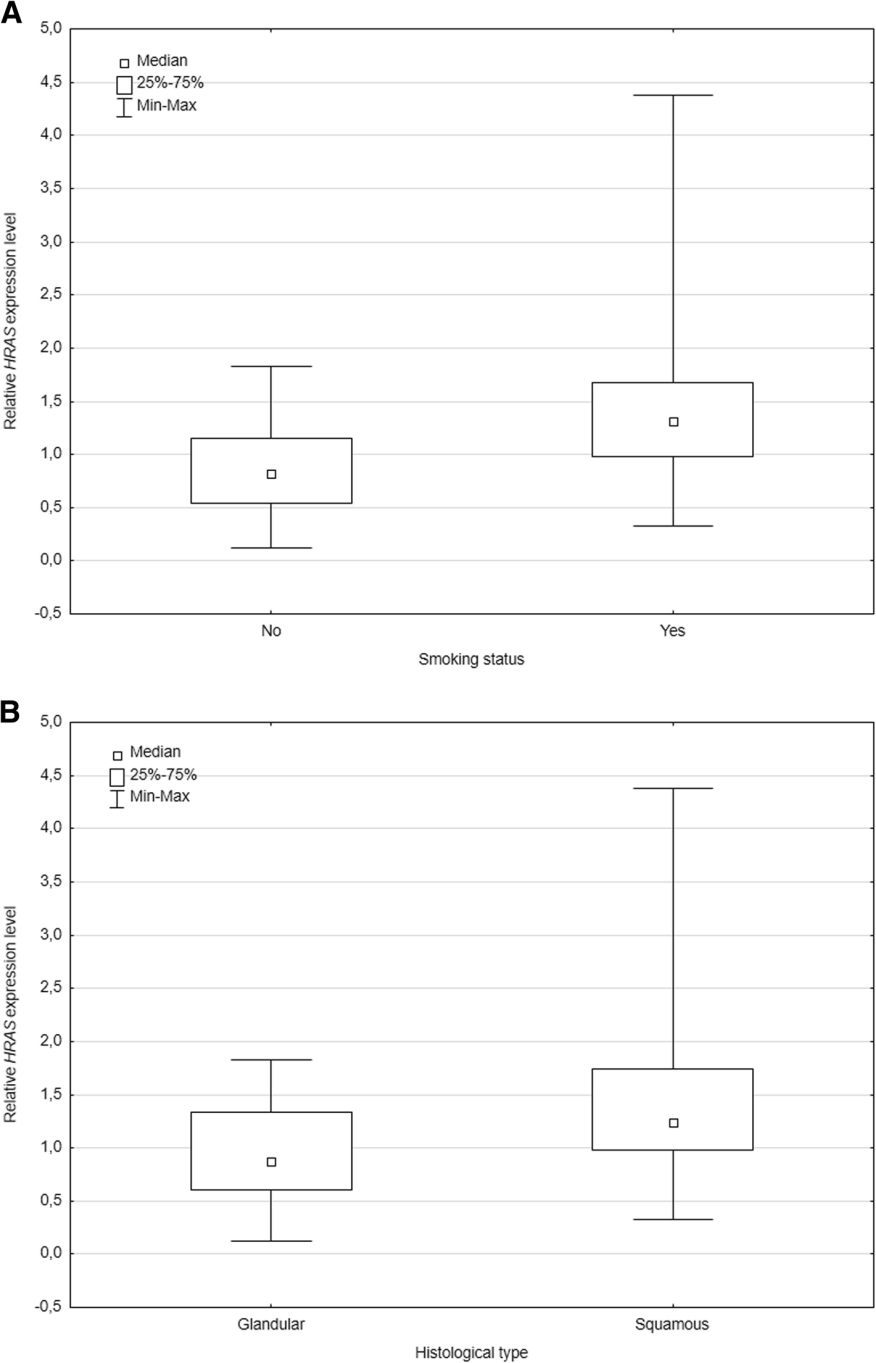
Relative *HRAS* gene expression level in blood of non-small-cell lung cancer cases. **a** shows the dependence of the relative *HRAS* gene expression level in relation to the smoking status of patients included in the study. **b** shows the relative *HRAS* gene expression level depending on the histological type of cancer. The data plots for each of the study groups represent the median value along with the minimum and maximum values and the lower and upper quartiles. Statistically significant differences were observed between smokers and non-smokers (*p =* 0.008), the differences were also noticeable but not statistically significant depending on histological type of cancer (*p =* 0.065)

The lung cancer cohort was divided into the subgroup of tobacco smokers (*N* = 25) and non-smokers (*N* = 14). Patients who were smokers had a significantly higher median relative for *HRAS* mRNA expression (*p =* 0.008). Data is summarized in Fig. 1a. Interestingly, such an association was not found for the relative *KRAS* mRNA expression.

Then, the investigated group was divided according to the histological type of cancer. Although the higher relative level of *HRAS* gene expression was observed in the group of patients with squamous cell carcinoma subtypes of NSCLC (*N* = 23) than in the group with adenocarcinoma (*N* = 16), this difference was not statistically significant (*p =* 0.065; Fig. 1b).

There were no associations between the relative *KRAS* mRNA expression and histological type, TNM stage, histological tumor grade or chemotherapy treatment. Similarly, no statistically significant correlations were found for TNM, histological tumor grade or chemotherapy treatment and the expression of *HRAS* mRNA.

### *Quantitative expression of the KRAS and HRAS genes in cancer tissue patients w*ith *NSCLC*

In 35 tumor samples extraction of mRNA was successful and all samples were included in the analysis of relative *KRAS* and *HRAS* mRNA expression levels using qRT-PCR. The median *KRAS* mRNA expression in tumor tissue obtained during surgery was 0.892 (range, 0.063–12.918), while the median *HRAS* mRNA expression was 0.978 (range, 0.054–24.194). Patients characteristics according to median relative *KRAS* and *HRAS* mRNA expression are shown in Table 3.

**Table 3:** Relative expression levels of *KRAS* (A) and *HRAS* (B) mRNA in tissue of NSCLC patients, compared to clinicopathological and demographical features

		Relative ***KRAS*** expression level in tissue				
		N	Median	Minimum	Maximum	***p***-value
A						
All cases		35	0.8917	0.0631	12.9178	
Cigarette	No	13	0.7730	0.0631	7.0357	1.0000
	Yes	22	0.9600	0.0941	12.9178	
Sexr	Women	7	0.7730	0.0941	8.2563	0.8528
	Men	28	0.9586	0.0631	12.9178	
Age	<= 67	18	0.3768	0.0941	3.5158	0.0361
	> 67	17	1.2411	0.0631	12.9178	
Histological type of cancer	Squamous	21	0.8917	0.0941	12.9178	0.5333
	Glandular	14	0.8652	0.0631	7.0357	
TNM stage	IA1 and/or IA2 and/or IB	16	0.9589	0.0631	12.9178	0.9868
	II A and/or II B and/or IIIA	19	0.7534	0.1109	8.2563	
Grade of histological malignancy [G]	G1 and/or G2	27	0.9575	0.0631	12.9178	0.5169
	G3	8	0.5918	0.1109	6.4671	
Chemotherapy	No	22	0.8045	0.0631	12.9178	0.1887
	Yes	13	0.9597	0.2825	7.0357	
B						
All cases		35	0.9779	0.0544	24.1938	
Cigarette	No	13	0.9779	0.0737	11.5889	0.7491
	Yes	22	0.8728	0.0544	24.1938	
Sex	Women	7	0.2131	0.0737	6.7824	0.3325
	Men	28	1.0848	0.0544	24.1938	
Age	<= 67	18	0.3621	0.0544	5.3788	0.0892
	> 67	17	1.2699	0.0653	24.1938	
Histological type of cancer	Squamous	21	0.4161	0.0544	24.1938	0.0489
	Glandular	14	1.6400	0.0797	11.5889	
TNM stage	IA1 and/or IA2 and/or IB	16	0.6167	0.0737	24.1938	0.6311
	II A and/or II B and/or IIIA	19	1.0854	0.0544	11.5889	
Grade of histological malignancy [G]	G1 and/or G2	27	0.6614	0.0737	24.1938	0.7683
	G3	8	1.2824	0.0544	11.5889	
Chemotherapy	No	22	0.4941	0.0544	24.1938	0.0978
	Yes	13	1.3379	0.1702	11.5889	

**Fig. 2:**
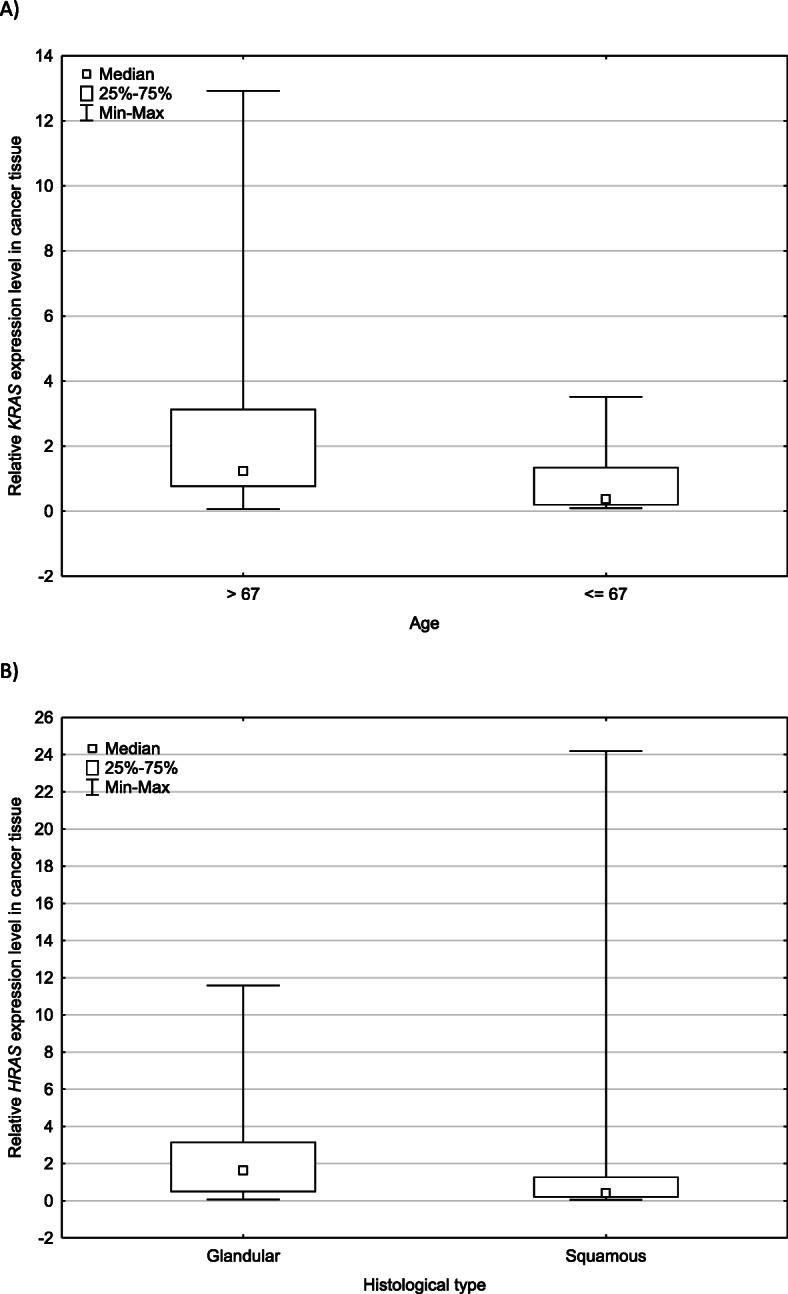
Relative *KRAS* and *HRAS* genes expression levels in tumor tissue of non-small-cell lung cancer cases. **a** shows the dependence of the relative *KRAS* gene expression level in relation to the age of patients included in the study. **b** shows the relative *HRAS* gene expression level depending on the histological type of cancer. The data plots for each of the study groups represent the median value along with the minimum and maximum values and the lower and upper quartiles. Statistically significant differences were observed depending on the age (*p =* 0.036) and histological type of cancer (*p =* 0.049)

Firstly, the examined cohort was divided into two groups according to age: 18 people under 67 years old and 17 people aged over 67 years of age. Statistically significant difference was observed between relative gene expression level and patients age (*p =* 0.036), the expression of *KRAS* gene was increased in the subgroup of patients over 67 years of age at diagnosis (Fig. 2a). The expression of the *HRAS* mRNA tended to be more expressed in the older group, but that was not statistically significant (*p* = 0.089).

Then, the lung cancer cohort was divided according to histological type of cancer. The subgroup of adenocarcinoma had significantly higher median relative *HRAS* mRNA expression level as compared with the subgroup of squamous cell carcinoma. (1.64 versus 0.416, *p* = 0.0489). Data is summarized in Fig. 2b.

The relative expression of the *HRAS* gene had a tendency to be more expressed in the subgroup who afterward received chemotherapy treatment, but that was not statistically significant (*p* = 0.098).

There were no associations between relative expression levels of *KRAS* gene and sex, histological type of cancer, smoking history, stage or implemented postoperative chemotherapy.

Likewise, no statistically significant correlations were found for smoking status, sex, TNM stage or histological tumor grade and the expression of *HRAS* mRNA.

### Comparison of the changes between the relative level of KRAS and HRAS genes expression in blood patients with NSCLC during a one-year observation at 3 points of time

**Fig. 3:**
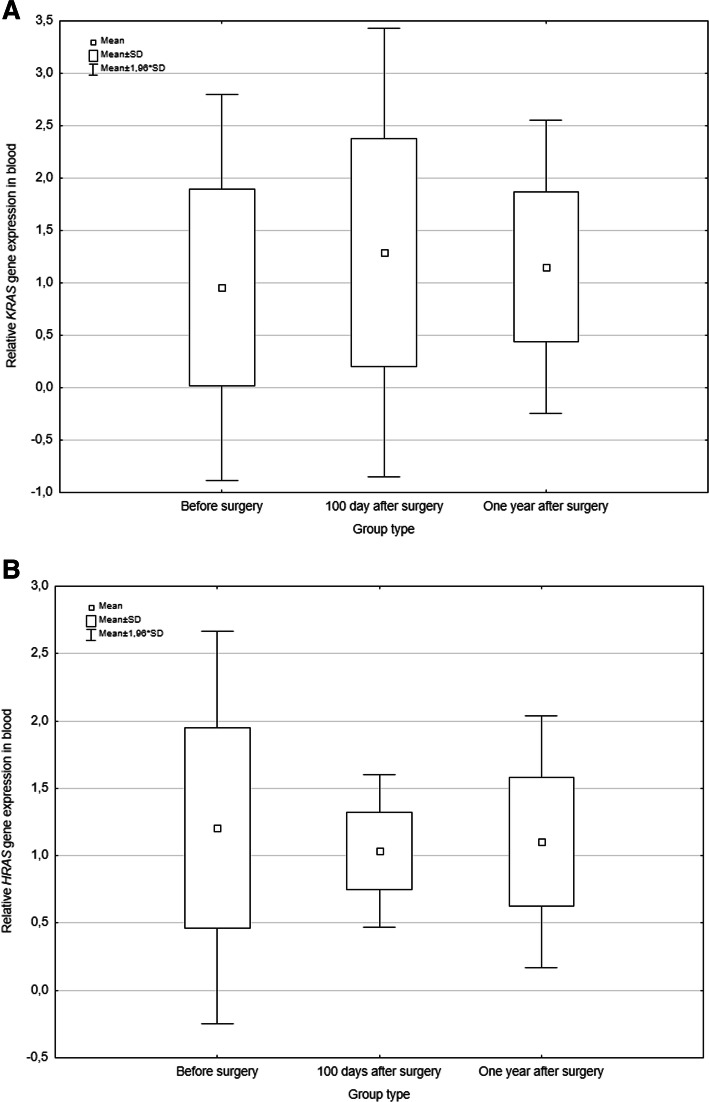
Relative *KRAS* and *HRAS* genes expression levels in blood of non-small-cell lung cancer cases during the observation. **a** shows the dependence of the relative *KRAS* gene expression levels and **b** illustrates the relative *HRAS* gene expression levels based on time in which the whole blood samples were collected: before the surgery, 100 days after the surgery and 1 year after the surgery. The data plots for each of study groups represent the mean value ± the standard deviation

Levels of investigated genes were checked in the duration of the disease which was clinically important. The changes of relative levels of relative *KRAS* and *HRAS* genes expression were assessed at three points of time (Fig. 3). There were no statistically significant differences between these groups (*p =* 0.766 for *KRAS*, *p =* 0.766 for *HRAS*).

### Comparison between the relative level of KRAS and HRAS genes expression in blood patients with NSCLC obtained before surgery and tissue samples taken during surgery

**Fig. 4:**
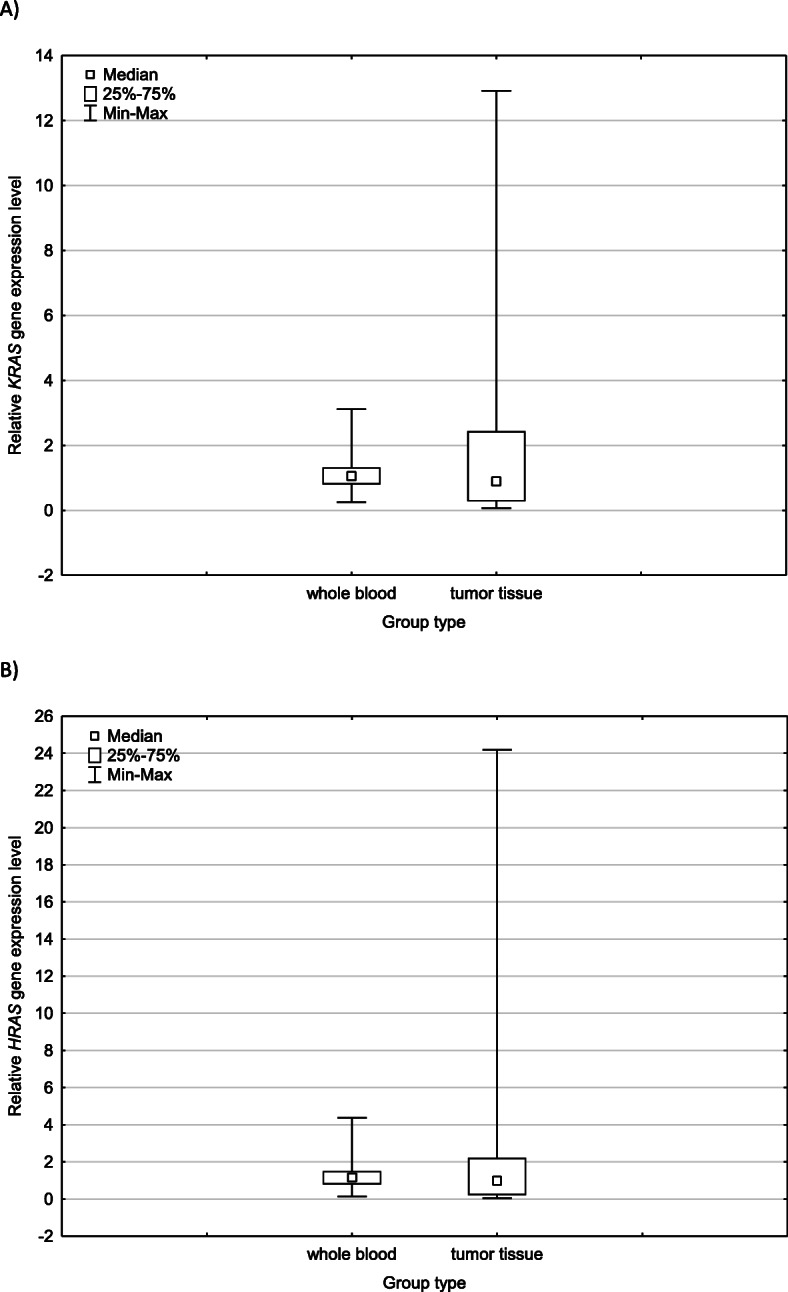
Relative expression levels of *KRAS* and *HRAS* in tumor tissue and blood taken from patients before surgery. **a** shows the dependence of the relative *KRAS* gene expression levels and **b** illustrates the relative *HRAS* gene expression levels between two types of NSCLC cohort materials. The data plots for each of study groups represent the median value along with the minimum and maximum values and the lower and upper quartiles

Relative levels of examined genes expression in the study cohort were compared between whole blood drawing before the surgery and tumor tissue samples obtained during the operation (Fig. 4). There were no statistically significant differences between the levels of *KRAS* and *HRAS* genes expression in tissue and blood before the surgery (*p =* 0.857 for *KRAS*, *p =* 0,935 for *HRAS*).

## Discussion

In the present study, relative *KRAS* and *HRAS* genes expression levels were retrospectively evaluated in 39 whole blood samples collected from patients with NSCLC at three points of time (at the time of diagnosis, 100 days and 1 year after the surgery) and in 35 tumor tissue samples obtained during operation using the real-time PCR method. To the best of our knowledge, this is the first such kind of study assessing the clinical role of mRNA levels of *KRAS* and *HRAS* in non-small-cell lung cancer cohort.

Some of the most important observations from the presented work were that overexpression of *HRAS* gene in blood had occurred more frequently in smokers and there was a tendency to the higher expression of the *HRAS* gene in patients with squamous cell carcinoma subtypes of NSCLC. Such association with smoking status was not observed for tumor tissue analysis. Moreover, considering results of expression in tissue samples, adenocarcinoma subgroup of patients had significantly higher median relative *HRAS* mRNA expression level. What is also interesting, the overexpression of *KRAS* gene in tissue was noticed in the subgroup of patients over 67 years of age at diagnosis. Similarly to this result, *HRAS* mRNA expression level was linked with the age of NSCLC cohort, but not statistically significant. Furthermore, *HRAS* gene had a tendency to overexpression in the subgroup who received postoperative chemotherapy. The data from the current study suggest that the expression of *HRAS* mRNA especially is worth further evaluation as a prognostic biomarker in lung cancer.

The three members of the *RAS* gene family (*HRAS, KRAS* and *NRAS*) are well-known because of their common oncogenic activation in human tumors, therefore they act as protooncogenes. The expression of *HRAS, KRAS* and *NRAS* genes is frequent in different species, although there are specific variations of expression levels, depending on the tissue and the developmental stage under study [10].

The majority of reported data focus on the frequency of *RAS* genes mutations. They indicate that *RAS* genes are often mutated in different types of tumor and participate in their proliferation apoptosis, migration and the differentiation of the cells. The *KRAS* mutation is common in smoking lung adenocarcinoma patients with frequency between 12 and 36%. *KRAS* mutation frequency in squamous cell lung carcinoma in smokers is 2.7%. The *HRAS* mutations are detected very rarely in lung cancers (< 1%) [11–13]. The prognostic value of *KRAS* and *HRAS* expression has been evaluated in various types of cancers so far, but only few studies indicated the association between the *RAS* family overexpression or mutation and the high degree lesions. For instance, Banys-Paluchowski et al. investigated the clinical significance of mRNA levels of the three RAS isoforms (*KRAS, HRAS* and *NRAS*) in a group of breast cancer patients. They found, among others, a correlation between overexpression of HRAS and the larger tumors, while increased *KRAS* levels were related to nodal positivity [14]. However, the expression of these oncogenes in different types of cancer tissue samples was mostly analyzed using immunohistochemistry. There are only few studies dedicated to *KRAS* and *HRAS* expression at mRNA level in lung cancer tissue [15–18].

To our knowledge, there is no research focusing on expression *RAS* genes determination in the whole blood assessed in Polish patients with NSCLC. Due to the limited data dedicated to the evaluation of the *RAS* genes expression in lung cancer, in current study the assessment of relative *KRAS* and *HRAS* gene expression level was performed. This research was aimed at considering the potential clinical usefulness *KRAS* and *HRAS* gene as tumor markers playing a role in the diagnosis (the differentiation between malignant and benign disease or histological subtype) as well as predictive biomarkers. The presented study showed that the relative expression of *HRAS* gene in blood patients with NSCLC was positively correlated with tobacco smoking. Taking into account the clinical characteristics of the investigated cohort more patients were smokers (64%), which correlates with the data from literature indicating that smoking is strongly associated with lung cancer risk. What is more, in the study group of NSCLC patients with squamous cell carcinoma, the majority of them were smokers. This finding is in agreement with literature review performed by Hirsch et al. described that squamous cell carcinoma has the strongest association with smoking, while adenocarcinoma is also associated with smoking, but there is a greater occurrence in non-smokers [19]. These data suggest that smoking is an important risk factors in lung cancer arise and could lead to overexpression of *HRAS* gene. Our results are similar to Krishna et al. findings which point out that the frequency of *HRAS* positive expression was higher in patients with oral squamous cell carcinoma who were smokers [17]. Such association between smoking status and gene expression was not found for *HRAS* gene expression in tumor tissue samples. Interestingly, the relative level of *HRAS* gene expression in blood was shown to be higher in the squamous cell carcinoma than in glandular subtypes, indicating that *HRAS* gene expression may be regulated developmentally and differentially. On the other hand, *HRAS* mRNA in tumor was overexpressed in adenocarcinoma. These inequalities in *HRAS* gene expression levels depending on the type of analyzed material might be explained by the fact that in squamous cell subtype of NSCLC, cells could be shed from the tumor and entered the bloodstream [20]. Due to this fact, genes expression detected in whole blood could be higher than in tissue. Unfortunately, it is not possible to compare these findings with other research because of the lack of evidence from previous study focusing on this issue. Another explanation for the significant differences in the *HRAS* gene expression level but not *KRAS* in NSCLC patients, seems the fact that oncogenic alterations in *KRAS* are more frequent in patients with lung malignancies [21]. Therefore, changes in *KRAS* expression might not be detectable, neither in blood nor in tumor tissue, except with observed by us the age-relative of *KRAS* gene expression in lung cancer tissue. Liang W. et al. showed that the expression of *KRAS* mRNA and protein was significantly increased in NSCLC compared the non-tumor tissues (*p = 0*.03 and *p =* 0.018, respectively). Moreover, the expression of KRAS protein was associated with tumor stages and also occurred more frequently in ever-smokers (*p =* 0.002) [22]. In this study no comparison between tumor and non-tumor tissue was performed, as it is hard to obtain a normal tissue without any lesions. Zhou et al. found that *KRAS* overexpression was common in acute myeloid leukemia patients and was associated with shorter overall survival [23]. In other study, Sugita et al. showed that *HRAS* expression levels were significantly upregulated in bladder cancer cell lines [24]. In this study, no significant associations between the *KRAS* mRNA expression in blood or tissue and clinicopathological parameters were observed. Wan et al. displayed an association between a lower *HRAS* expression level with longer overall survival in cutaneous melanoma [25]. Furthermore, An et al. showed *RAS* as an independent predictor of overall survival in patients with lung cancer who were treated with bevacizumab and chemotherapy. They revealed that a lower *RAS* expression level was associated with longer survival time [16]. Similarly, Chen et al. revealed *KRAS* overexpression in patients with colorectal cancer and the high expression of *KRAS* predicted poor treatment outcomes in patients. However, they analyzed the protein level of KRAS in tissue samples using immunohistochemistry [26]. We are conscious that larger sample cohort would be needed in this study to notice survival differences in correlation with genes expression. Therefore, verifying the impact of *KRAS* and *HRAS* genes expression levels on the survival NSCLC patients would be the next aim of our study.

In spite of the fact that *RAS* isoforms share related downstream pathways, their posttranscriptional regulation may differ from each other [25]. Our study suggests that regulation of *HRAS* in non-small-cell lung carcinoma vary from that of *KRAS*. RAS isoform differences have been identified at the level of protein translation and provide a potential explanation for the highest frequency of *KRAS* mutation in cancers. *KRAS* DNA coding sequence has a high frequency of rare codons, causing poor KRAS protein translation and expression, which is in contrast to *HRAS*. It has been suggested that the overexpression of *HRAS*, but not *KRAS*, induces senescence. Therefore, a cell with mutated *KRAS* will persist to allow succeeding genetic events to promote tumor progression [27], which could be an explanation as for why we did not notice statistically significant changes in the *KRAS* gene expression. To observe the possible dynamic changes of *KRAS* and *HRAS* expression in NSCLC patients at different clinical stages, *RAS* expression levels were assessed at three points of time during the follow-up studies. Neither *KRAS* nor *HRAS* expression tended to fluctuate for the time of a 1-year observation compared to newly diagnosis time. Both *KRAS* and *HRAS* gene expression were not significantly increased or decreased between the first assessment, 100 days and 1 year after the diagnosis. Consequently, they could not be used as markers of the disease progression or the effectiveness of the therapy. As Liang et al. have noticed, the differential expression of genes in NSCLC suggests the presence of a complex regulatory network involving tumor suppression and oncogenic expression [22]. Therefore, it is worth conducting a further investigation. In the present study, there were no statistically significant differences between the levels of *KRAS* and *HRAS* genes expression in tissue and blood before the surgery. Therefore, it could be hypothesized that expression values in tissue and blood do correlate. We are aware of the limitations of the current research, such as small the investigated group from a single institution and do not carry out survival analysis, but the results described in this paper are preliminary. Future studies will include a larger cohort of NSCLC patients with longer follow-ups and multicenter study to verify the obtained findings. The next step of the analysis will be extended to the evaluation of the impact of *KRAS* and *HRAS* genes expression on the overall survival.

## Conclusions

Overexpression of *HRAS* gene was observed in the blood group of smokers and tended to be more presumable in squamous cell lung cancer, while *HRAS* mRNA expression in tissue was increased in adenocarcinoma. The higher levels of *HRAS* and *KRAS* genes expression was observed in NSCLC patients over 67 years of age at diagnosis. This study did not reveal statistically significant differences between the relative expression level of *KRAS* and *HRAS* genes in NSCLC patients at three points of time during the observation, thus it is not possible to use them as markers of the disease progression. Moreover, relative levels of *KRAS* and *HRAS* genes expression in tissue and blood before the surgery had no statistical differences. This study emphasized the role of *HRAS* and *KRAS* gene expression levels and their heterogeneity in non-small-cell lung cancer cases. It may improve our knowledge for a better diagnosis, identifying patients at high risk of a disease and finding new predictive biomarkers.

## Supplementary Information

**Additional file 1: Supplementary Table 1.** Detailed clinicopathological and laboratory characteristics each of NSCLC patients.

## Data Availability

The datasets used and/or analyzed during the current study are available from the corresponding author on reasonable request.

## Abbreviations

GAPDH: Glyceraldehyde-3-phosphate dehydrogenase
HRAS: Harvey rat sarcoma viral oncogene homolog
KRAS: Kirsten rat sarcoma viral oncogene homolog
NLR: Neutrophil to Lymphocyte Ratio
LMR: Lymphocyte to Monocyte Ratio
NRAS: neuroblastoma RAS viral oncogene homolog
NSCLC: Non–small-cell lung cancer
OS: Overall survival
PLR: Platelet to Lymphocyte Ratio
PLT: Platelets
RBC: Red blood cells
TNM: Tumor-node-metastasis
WBC: White blood cells
