# Supplementary material for: Clinical significance of HRAS and KRAS genes expression in patients with non–small-cell lung cancer - preliminary findings
Source: BMC Cancer. 2021 Feb 6;21:130. doi: 10.1186/s12885-021-07858-w (PMC7866659; doi:10.1186/s12885-021-07858-w)
Supplement: Supplementary file 1 — Additional file 1: Supplementary Table 1. Detailed clinicopathological and laboratory characteristics each of NSCLC patients. [file 12885_2021_7858_MOESM1_ESM.docx]

| Patient | Smoking | Pack Years of Smoking | Histological type of cancer | TNM stage | Grade of histological malignancy [G] | Chemotherapy | WBC (x 10^3^/µl) | RBC (x 10^6^/µl) | PLT (x 10^3^/µl) | NLR | LMR | PLR |
| --- | --- | --- | --- | --- | --- | --- | --- | --- | --- | --- | --- | --- |
| 1 | no | N/A | adenocarcinoma | IIIA | G2 | cisplatin, vinorelbine | 7.31 | 4.55 | 250 | 5.80 | 1.26 | 268 |
| 2 | yes | 40 | squamous cell | IB | G2 | No | 10.33 | 4.95 | 317 | 3.07 | 2.80 | 140 |
| 3 | no | N/A | adenocarcinoma | Ia1 | G1 | No | 7.27 | 4.86 | 258 | 2.49 | 4.97 | 132 |
| 4 | yes | 50 | squamous cell | IIA | G3 | No | 13.00 | 4.05 | 372 | 4.73 | 1.25 | 192 |
| 5 | no | N/A | adenocarcinoma | IA2 | G3 | No | 14.70 | 3.54 | 536 | 3.06 | 5.87 | 147 |
| 6 | yes | 40 | squamous cell | IB | G2 | No | 9.07 | 3.55 | 215 | 1.22 | 2.90 | 62 |
| 7 | yes | 40 | squamous cell | IIIA | G2 | cisplatin, vinorelbine | 9.43 | 4.66 | 169 | 5.35 | 1.37 | 128 |
| 8 | no | N/A | adenocarcinoma | IIIA | G3 | carboplatin, gemcitabine | 10.36 | 3.96 | 301 | 4.31 | 3.08 | 165 |
| 9 | yes | 30 | adenocarcinoma | IIIA | G2 | cisplatin, vinorelbine | 15.76 | 5.11 | 382 | 1.99 | 0.68 | 82 |
| 10 | yes | 20 | squamous cell | IIIA | G2 | cisplatin, vinorelbine | 11.44 | 4.73 | 356 | 5.50 | 1.28 | 235 |
| 11 | yes | 45 | adenocarcinoma | IIB | G1 | carboplatin, gemcitabine | 10.98 | 4.61 | 329 | 3.07 | 2.20 | 139 |
| 12 | yes | 40 | adenocarcinoma | IB | G2 | No | 13.07 | 4.57 | 352 | 5.06 | 1.89 | 182 |
| 13 | yes | 40 | adenocarcinoma | IA2 | G2 | No | 6.68 | 4.68 | 364 | 1.85 | 4.28 | 173 |
| 14 | yes | 30 | squamous cell | IIB | G2 | cisplatin, vinorelbine | 9.47 | 3.87 | 299 | 1.86 | 2.77 | 107 |
| 15 | yes | 40 | squamous cell | IIIA | G2 | cisplatin, vinorelbine | 9.31 | 5.18 | 208 | 1.49 | 5.61 | 61 |
| 16 | no | N/A | squamous cell | IB | G2 | No | 10.64 | 4.33 | 290 | 8.33 | 1.38 | 207 |
| 17 | yes | 20 | squamous cell | IA2 | G2 | No | 10.20 | 4.08 | 475 | 2.09 | 4.78 | 158 |
| 18 | no | N/A | adenocarcinoma | IB | G2 | No | 7.48 | 5.00 | 190 | 3.70 | 2.24 | 137 |
| 19 | yes | 30 | squamous cell | IIA | G2 | No | 10.67 | 5.08 | 237 | 4.97 | 1.34 | 152 |
| 20 | yes | 40 | squamous cell | IIA | G3 | No | 19.39 | 3.81 | 617 | 10.50 | 1.38 | 392 |
| 21 | no | N/A | adenocarcinoma | IIB | G3 | cisplatin, vinorelbine | 6.88 | 5.09 | 259 | 1.54 | 2.92 | 112 |
| 22 | yes | 30 | squamous cell | IIA | G2 | No | 8.98 | 4.15 | 300 | 5.76 | 1.36 | 256 |
| 23 | yes | 40 | adenocarcinoma | IIB | G2 | cisplatin, vinorelbine | 15.11 | 5.84 | 961 | 6.96 | 0.62 | 154 |
| 24 | yes | 40 | squamous cell | IIA | G3 | No | 9.65 | 4.33 | 273 | 3.83 | 2.53 | 152 |
| 25 | no | N/A | squamous cell | IB | G2 | No | 8.48 | 4.05 | 263 | 4.76 | 1.79 | 207 |
| 26 | no | N/A | squamous cell | IB | G2 | No | 8.26 | 4.56 | 235 | 4.01 | 2.17 | 340 |
| 27 | yes | 40 | squamous cell | IB | G3 | No | 8.83 | 5.02 | 267 | 2.75 | 1.67 | 133 |
| 28 | no | N/A | adenocarcinoma | IIIA | G3 | carboplatin, gemcitabine | 7.05 | 3.72 | 272 | 4.43 | 2.35 | 555 |
| 29 | no | N/A | squamous cell | IIB | G2 | cisplatin, vinorelbine | 8.53 | 4.73 | 301 | 2.58 | 3.43 | 141 |
| 30 | no | N/A | adenocarcinoma | IIA | G2 | cisplatin, vinorelbine | 6.02 | 4.32 | 508 | 5.66 | 1.63 | 976 |
| 31 | no | N/A | adenocarcinoma | IA2 | G1 | No | 6.55 | 4.69 | 172 | 2.05 | 4.17 | 88 |
| 32 | yes | 40 | squamous cell | IB | G2 | No | 10.35 | 4.18 | 167 | 10.91 | 0.89 | 211 |
| 33 | yes | 50 | adenocarcinoma | IIA | G2 | No | 14.29 | 3.93 | 434 | 5.36 | 1.49 | 215 |
| 34 | yes | 50 | squamous cell | IB | G2 | No | 12.73 | 4.11 | 201 | 20.39 | 0.78 | 358 |
| 35 | yes | 40 | squamous cell | IA2 | G2 | No | 6.50 | 5.07 | 228 | 1.92 | 3.58 | 116 |
| 36 | yes | 40 | squamous cell | IIIA | G2 | No | 12.77 | 5.02 | 432 | 3.88 | 3.08 | 177 |
| 37 | yes | 40 | squamous cell | IA2 | G2 | No | 10.91 | 5.24 | 329 | 3.22 | 1.95 | 144 |
| 38 | yes | 40 | squamous cell | IB | G2 | No | 11.15 | 4.86 | 310 | 0.97 | 4.91 | 64 |
| 39 | no | N/A | adenocarcinoma | IA2 | G2 | No | 7.80 | 4.92 | 278 | 4.57 | 2.44 | 215 |

**Abbreviations:** N/A, not applicable; NLR, Neutrophil to Lymphocyte Ratio; LMR, Lymphocyte to Monocyte Ratio; PLR, Platelet to Lymphocyte Ratio; TNM, tumor-node-metastasis; WBC, white blood cells; RBC, red blood cells; PLT, platelets
